# Endothelial Cell and Platelet Bioenergetics: Effect of Glucose and Nutrient Composition

**DOI:** 10.1371/journal.pone.0039430

**Published:** 2012-06-22

**Authors:** Brian D. Fink, Judy A. Herlein, Yunxia O’Malley, William I. Sivitz

**Affiliations:** 1 Department of Internal Medicine/Endocrinology and Metabolism, University of Iowa Hospitals and Clinics and Iowa City VAMC, Iowa City, Iowa, United States of America; 2 Department of Surgery, University of Iowa Hospitals and Clinics and Iowa City VAMC, Iowa City, Iowa, United States of America; University of South Alabama, United States of America

## Abstract

It has been suggested that cells that are independent of insulin for glucose uptake, when exposed to high glucose or other nutrient concentrations, manifest enhanced mitochondrial substrate oxidation with consequent enhanced potential and generation of reactive oxygen species (ROS); a paradigm that could predispose to vascular complications of diabetes. Here we exposed bovine aortic endothelial (BAE) cells and human platelets to variable glucose and fatty acid concentrations. We then examined oxygen consumption and acidification rates using recently available technology in the form of an extracellular oxygen and proton flux analyzer. Acute or overnight exposure of confluent BAE cells to glucose concentrations from 5.5 to 25 mM did not enhance or change the rate of oxygen consumption (OCR) under basal conditions, during ATP synthesis, or under uncoupled conditions. Glucose also did not alter OCR in sub-confluent cells, in cells exposed to low serum, or in cells treated with added pyruvate. Likewise, overnight exposure to fatty acids of varying saturation had no such effects. Overnight exposure of BAE cells to low glucose concentration decreased maximal uncoupled respiration, but not basal or ATP related oxygen consumption. Labeled glucose oxidation to CO_2_ increased, but only marginally after high glucose exposure while oleate oxidation to CO_2_ decreased. Overnight exposure to linolenic acid, but not oleic or linoleic acid increased extracellular acidification consistent with enhanced glycolytic metabolism. We were unable to detect an increase in production of reactive oxygen species (ROS) from BAE cells exposed to high medium glucose. Like BAE cells, exposure of human platelets to glucose did not increase oxygen consumption. As opposed to BAE cells, platelet mitochondria demonstrate less respiratory reserve capacity (beyond that needed for basal metabolism). Our data do not support the concept that exposure to high glucose or fatty acids accelerates mitochondrial oxidative metabolism in endothelial cells or platelets.

## Introduction

Some have suggested that high circulating glucose concentrations delivered to cells that are independent of insulin for glucose entry leads to increased substrate delivery to mitochondria. Substrate oxidation would then increase membrane potential leading to enhanced mitochondrial superoxide production, thus, contributing to the long term complications of diabetes. Indeed, some studies of non-insulin-dependent cultured cells or *ex vivo* blood platelets reported increased reactive oxygen species (ROS) production as a result of exposure to high glucose in the media [1], [2], [3], [4]. In contrast, other reports show no such change in ROS [5], [6]. Moreover, there are reports of increased ROS production on exposure to low glucose [7], [8].

Notwithstanding the controversy regarding ROS, the underlying supposition that exposure of non-insulin dependent cells to glucose actually alters mitochondrial substrate oxidation has not been established. This issue cannot be addressed by studying mitochondria isolated after exposure to varied nutrient composition since the organelles are removed to a completely different and artificial extra mitochondrial environment. However, recent advances in technology have improved our capacity to assess oxidative metabolism in intact cells [9], [10]. Here we use this methodology to directly assess the effect of acute and chronic (overnight) glucose and fatty acid exposure on mitochondrial oxygen consumption by cultured vascular endothelial cells. We also examined the effect of acute glucose on mitochondrial oxidation in freshly isolated platelets obtained from non-diabetic and hyperglycemic, type 1 diabetic human subjects. Both endothelial cells and platelets are independent of insulin for glucose uptake and, therefore, potentially vulnerable to excess substrate delivery when exposed to high medium nutrient composition. Both cell/particle types are important in mediating the effects of glycemia on vascular function [11], [12] and abnormal endothelial function is a well-established risk factor for the macrovascular complications of diabetes [13].

Here, we show that endothelial cells and platelets remain robust in regard to mitochondrial oxidative metabolism, in spite of differences in glucose and fatty acid exposure. We also identify some contrasts between the bioenergetic properties of endothelial cells and platelets and between fatty acids of differing saturation.

## Methods

### Human Subjects Declaration

These studies were approved by the University of Iowa Institutional Review Board (IRB). All participants signed an IRB approved written informed consent and all studies were conducted according to the principles expressed in the Declaration of Helsinki.

### Reagents and Supplies

Reagents, kits, and supplies were obtained as specified or purchased from standard sources.

### Cell Culture

BAE cells were grown in medium M199 (Invitrogen) supplemented with MEM non-essential amino acids (Invitrogen), MEM vitamins (Sigma), 2 mM L-glutamine (Invitrogen), 1 mM sodium pyruvate (Invitrogen), and 17% fetal bovine serum (HyClone, Logan, UT, USA) as described [14]. Cells were cultured in 75-cm^2^ flasks and were split at a 1∶10 ratio prior to reaching confluence. Cells were used between passages 5 and 10. For extracellular flux experiments (see below) split cells were seeded in 24-well respirometry plates (Seahorse Bioscience, North Billerica, MA, USA).

### Human Studies

Blood samples for platelets were obtained at 10–12 AM from five male and five female subjects with type 1 diabetes (age 43±4 years, BMI 27.6±2.2, HbA1c 7.8±0.4 corresponding average glucose 9.8±0.6 mM, and plasma glucose at blood draw 10.3±1.4 mM) diagnosed by an Endocrinologist and followed in our University Diabetes Out-Patient Clinic. Samples were also obtained from five female non-diabetic, healthy individuals (age 45±9 years, BMI 26.9±1.5, plasma glucose at blood draw 4.0±0.5 mM).

Subjects were included based on: 1) Age 18–70; 2) (for diabetic subjects) Type 1 diabetes based on typical history as assessed by an Endocrinologist and history of diabetic ketoacidosis or continuing need for insulin therapy since diagnosis or C-peptide less than 0.4 ng/100 ml in spite of glucose >6.7 mM. Subjects were excluded based on: 1) History of blood or platelet disorder; 2) Mitochondrial disorder; 3) Any other acute or chronic disease felt to interfere with data interpretation.

### Platelet Isolation

Platelet rich plasma (PRP) was obtained from 60 ml EDTA treated human blood using standard methods [15]. PRP was prepared by centrifugation at 120×*g* for 10 min at room temperature. PRP was then centrifuged at 750×g for 10 min to separate platelets from plasma. Platelets were washed at 750×*g* in the presence of 0.12 M NaCl, 3 mM Na EDTA, 5.5 mM D-glucose, and 0.03 M Tris HCl, pH 7.4, and counts were obtained using a Z1 Coulter Particle Counter (Beckman Coulter). This procedure generated 10^8^ to 10^9^ platelets per ml in a 5 ml volume.

### Respirometry (Extracellular Flux Analysis)

Oxygen consumption rate (OCR) and extracellular acidification rate (ECAR) were measured using an intact cell respirometer designed for adherent cells (XF-24 Extracellular Flux Analyzer, Seahorse Bioscience) [10]. BAE cells were seeded at a density of 5,000–10,000 cells per well in 24 well plates designed for respirometer analyses. Cells reached confluency after two days and were subjected to respirometry 3 to 4 days after seeding. Freshly isolated platelets were added to 24-well Seahorse plates and spun at 1000 g for 15 min immediately before respirometry assay.

**Figure 1:**
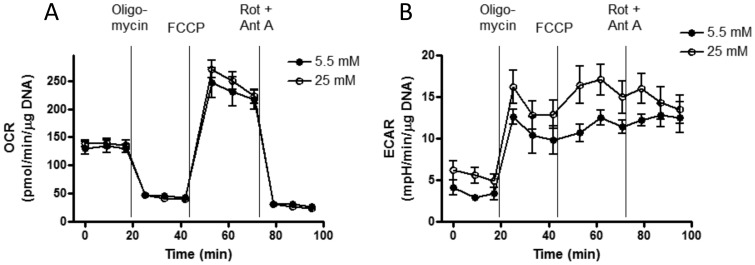
Oxygen consumption (OCR) and simultaneous rates of extracellular acidification (ECAR) by BAE cells exposed to differing glucose concentrations. Cells were exposed to 5.5 (closed circles) or 25 (open circles) mM glucose for 18 h prior to the experimental run with the same concentrations continued during the run. A) Representative experiment depicting OCR determined before (beginning time 0) and after sequential injections of the indicated compounds; oligomycin (2 µM), FCCP (2 µM), or antimycin A (Ant A, 0.5 µM) plus rotenone (Rot, 2 µM). B) Simultaneous ECAR measurements. Data show the mean ± SEM of 5 values determined for each data point for each glucose condition. Quantitative results of this and other experiments are listed in tables 1 and 2.

OCR and ECAR were determined in assay medium consisting of medium M199 lacking serum, bicarbonate, and pyruvate (Invitrogen) over time periods up to 95 minutes with assessments at 8–10 minute intervals. Where indicated under “results”, we carried out certain experiments wherein the Seahorse assay medium was supplemented with 25 mM HEPES, pH 7.4. Prior to analysis, cells within individual wells were exposed for 18 h to glucose or fatty acids as described in the legends and/or text. During respirometry, wells were sequentially injected (as exemplified in figure 1) at the times indicated in the figures with: oligomycin (2 µM) to block ATP synthase to assess respiration required for ATP turnover (OCR_ATP_); carbonyl cyanide p-[trifluoromethoxy]-phenyl-hydrazone (FCCP, 2 µM), a proton ionophore, to induce chemical uncoupling and maximal respiration (OCR_MAX_); or antimycin-A (0.5 µM) plus rotenone (2 µM) to completely inhibit electron transport and measure non-mitochondrial respiration. The FCCP concentration used in these studies was determined by titration with differing amounts of the uncoupler using the least amount required for maximal uncoupling. The above concentration of oligomycin, FCCP, antimycin, and rotenone apply to all figures in this manuscript.

All values for OCR and ECAR in BAE cells were normalized to DNA content of the individual wells. OCR and ECAR values for platelets were expressed per well (20×10^6^ platelets/well). Calculations of respiratory parameters were as described [16]. To calculate these parameters, we considered basal OCR as the last value (for example, see figure 1) prior to injection of the first additive (oligomycin). Likewise, we considered OCR on oligomycin as the last value prior to FCCP injection, OCR on FCCP as the last value prior to antimycin plus rotenone injection, and non-mitochondrial OCR as the last value recorded after antimycin plus rotenone.

As described [16], we then calculated the following parameters. OCR_BASAL_ was determined as OCR in the basal state minus non-mitochondrial OCR. OCR_ATP_ was determined as OCR in the basal state minus OCR on oligomycin. OCR_LEAK_ (in other words rate of oxygen consumption accountable by the proton leak) was calculated as OCR on oligomycin minus non-mitochondrial OCR. OCR_MAX_ was calculated as OCR on FCCP minus non-mitochondrial OCR. As described [16], “state apparent” was calculated as 4-[(OCR_ATP_)/(OCR on FCCP - OCR on oligomycin)]. State apparent provides an index of the respiratory state (extent of state 3 versus state 4 respiration as commonly defined for isolated mitochondria). ECAR (mpH/min) was quantified as the recorded acidification rates in the basal state and after injection of additives as described for the OCR measurements. ECAR is expressed as milli pH (mpH) units representing the change in pH per min where mpH = 1/1000^th^ pH unit.

### Quantification of DNA

After respirometry, well contents were extracted in 0.4% SDS and diluted to 0.01%. DNA content of each well was determined after respirometry using a Sigma DNA Quantitation Kit (DNA-QF) using calf thymus DNA standards prepared in 0.01% SDS.

### Glucose and Oleate Oxidation to CO_2_

Cells were grown in culture medium and exposed to 5.5 or 25 mM glucose for 18 h. Cells were then washed and pre-incubated for 20 min in culture medium with 20 µM oleate and 5.5 or 25 mM glucose with 1.5% fatty acid-free BSA, and 1 mM carnitine in 12-well plates (Costar, Corning Inc., Acton, MA) containing 1.0 ml total volume per well. In each experiment, glucose and oleate oxidation were assessed in parallel studies under the same conditions except for the addition of either [1–14C]oleic acid or D-[14C(U)]glucose. Cells were incubated for 120 min before trapping of CO_2_ released by perchloric acid as we previously described [17]. Final specific activities in the incubation media were 20.0 µCi/µmol for oleate and 0.36 µCi/µmol for glucose at 5.5 mM and 0.080 µCi/µmol for glucose at 25 mM.

### Glucose and Glutamine Oxidation to CO_2_

Cells were grown in culture medium and exposed to 5.5 or 25 mM glucose for 18 h plus supplemental glutamine to a final concentration of 2.7 mM. Cells were then washed and pre-incubated for 20 min in culture medium with 2.7 mM glutamine and 5.5 or 25 mM glucose with 1.5% fatty acid-free BSA, and 1 mM L-carnitine in 12-well plates and trapped CO_2_ was determined as above. Glucose and glutamine oxidation were assessed in parallel studies under the same conditions except for the addition of either [14C(U)]-glutamine or D-[14C(U)]glucose. Final specific activities in the incubation media were 0.19 µCi/µmol for glutamine and 0.36 µCi/µmol for glucose at 5.5 mM and 0.080 µCi/µmol for glucose at 25 mM.

### ROS Production

Oxidation of mitochondrial-targeted hydroethidine (MitoSOX, Invitrogen) was assessed by high performance liquid chromatography (HPLC) following a modification of reported methodology [18], [19]. Cells were washed twice with 2 mL of Dulbecco’s Phosphate Buffered Saline, containing calcium chloride and magnesium chloride (DPBS) followed by addition of 0.9 ml Earle’s Balanced Salt Solution (EBSS) containing glucose concentrations equivalent to prior exposures. Cells were then loaded with MitoSOX (10 µM), incubated for 20 min at 37°C, washed with DPBS, and further incubated for 60 min at 37°C with the same glucose concentrations with or without added antimycin A. Incubations were then stopped by addition of ice-cold DPBS after which cells were scrapped and suspended in small Eppendorf tubes, pelleted at 5 min x 1000 g and stored at −80°C. Subsequently, cells were lysed in 0.1% Triton X-100. Protein was determined on the lysates followed by extraction in methanol containing 0.2 M perchloric acid followed by extraction with 1 M potassium phosphate buffer, pH 2.8. HPLC was performed using a Supelco Ascentis C-18, 5 uM pore size, 25 cm×4.6 mM column at a flow rate of 1 ml/min with an injection volume of 50 µl. A mobile gradient consisted of 0.1% trifluoroacetic acid (TFA) in water (phase A) and 0.1% TFA in acetonitrile (phase B). Initial flow consisted of 20%B/80%A. Separation of peaks was achieved by a linear gradient of mobile phase B from 45 to 50% from time 10 to 20 minutes. Fluorescent detection was accomplished using excitation set at 510 nm and emission at 595 nm. UV detection was performed at 290 nm.

Fluorescent detection: H_2_O_2_ production was also measured using the fluorescent probes 10-acetyl-3,7-dihydroxyphenoxazine (DHPA or Amplex Red, Invitrogen) and 2',7'-dichlorodihydrofluorescein diacetate (H_2_DCFDA, Invitrogen) according to manufacturer recommendations. BAE cells were exposed to variant nutrient composition for 18 h as in the respirometry experiments. H_2_O_2_ production was measured with a microplate fluorescence reader using wavelength settings of 485 nm excitation and 520 nm emission for H_2_DCFDA or 544 nM excitation and 590 nm emission for DHPA. Assays were performed in medium M199 without serum, sodium bicarbonate, or pyruvate and containing 5 mM or 25 mM glucose.

Oxidative damage was also assessed as aconitase inactivation in isolated BAE mitochondria. Mitochondria of good quality and purity were isolated from BAE cells as we previously described [20]. Aconitase activity was measured according to a standard assay as NADPH at 340 nm [21]. Mitochondria were incubated as described [21] in assay buffer containing 2 units of isocitrate dehydrogenase, and 5 mM citrate, pH 7.2.

### Statistics

Data were analyzed by two-tailed, unpaired t-test or ANOVA as indicated in the figure legends, tables, or text.

## Results

### Acute and Chronic Glucose Exposure does not Increase Mitochondrial Oxygen Consumption by BAE Cells

Figure 1A depicts a representative experiment demonstrating the effect of overnight exposure to 5.5 or 25 mM glucose on basal OCR and on OCR as affected by sequential additions of oligomycin, FCCP, and rotenone plus antimycin A. The overnight glucose concentrations were maintained during the experimental run. Glucose exposure had no significant effects on OCR under any of the conditions examined. Table 1 lists the results of multiple repetitions of the same experiment confirming the lack of effect of perturbed glucose concentrations.

**Table 1:** Effect of chronic exposure to glucose on oxygen consumption rates (OCR) and extracellular acidification rates (ECAR) in BAE cells exposed to concentrations ranging from 5.5 to 25 mM for 18 h with these concentrations maintained during incubation in the extracellular flux analyzer.

D-glucose (mM)	5.5	11	18	25	5.5+19 mM L-glucose
OCR (pmol/min/µg DNA)					
OCR_BASAL_	79±7	88±9	89±11	73±14	84±15
OCR_ATP_	68±5	75±5	74±9	61±11	72±12
OCR_MAX_	127±19	144±23	152±32	113±23	132±23
Non-mito	25±3	29±4	27±4	24±4	28±5
OCR_LEAK_	11±4	13±4	16±3	12±3	12±4
ECAR (mpH/min/µg DNA)					
Basal	11.0±1.8	10.0±1.6	12.9±1.8	11.4±1.6	10.7±1.6
Oligomycin	24.1±3.6	24.1±3.6	30.1±4.5 *	29.4±4.4 *	25.2±3.9
FCCP	29.1±3.9	27.9±3.4	32.9±4.2	31.6±3.6	28.6±4.7
Non-mito	30.8±4.4	31.5±4.5	35.2±5.6	33.9±4.9	29.5±5.0

* p<0.05 versus 5.5 mM glucose condition by repeated measures one-way ANOVA, n = 6 experiments with each individual value representing a mean of 4–5 wells within a single experimental run.

Similar data were obtained when cells were maintained at 5.5 mM glucose overnight followed by acute exposure to variant glucose added at the onset of each experimental run (table 2).

**Table 2:** Effect of acute exposure to glucose on oxygen consumption rates (OCR) and extracellular acidification rates (ECAR) in BAE cells exposed to concentrations of 5.5 mM to 25 mM for 20 minutes with these concentrations maintained during incubation in the extracellular flux analyzer, n = 6 experiments with each individual value representing a mean of 4–5 wells within a single experimental run.

D- glucose (mM)	5.5	11	18	25	5.5+19 mM L-glucose
OCR (pmol/min/µg DNA)					
OCR_BASAL_	90±15	90±13	80±11	80±10	81±10
OCR_ATP_	63±12	72±10	62±8	62±7	63±7
OCR_MAX_	147±31	144±32	140±33	127±32	142±33
Non-mito	22±11	25±9	28±10	29±10	30±9
OCR_LEAK_	27±7	19±4	18±4	19±4	18±3
ECAR (mpH/min/µg DNA)					
Basal	14.5±4.5	17.1±5.2	16.3±4.2	16.7±4.3	15.8±4.5
Oligomycin	26.2±8.1	27.5±8.6	28.0±7.3	28.9±8.0	24.4±6.7
FCCP	28.2±8.6	27.5±9.5	25.4±6.5	26.9±8.1	24.4±6.6
Non-mito	29.2±9.1	28.4±9.1	27.8±7.3	27.6±8.8	24.3±6.8

No significant differences were observed.

### Effect of Acute and Chronic Glucose Exposure on Extracellular Acidification Rates in BAE Cells

As shown in table 2, acute exposure to high glucose had no significant effect on ECAR. However, as seen in figure 1B and table 1, chronic exposure to 18 mM or 25 mM glucose compared to 5.5 mM glucose did increase ECAR after oligomycin injection. There was also a trend towards glucose-induced increased ECAR during basal respiration, after FCCP, and when mitochondrial electron transport was blocked by antimycin plus rotenone (table 1).

### Effect of Glucose Exposure on OCR and ECAR under Variant Cell Nutrient and Growth Conditions

We also examined mitochondrial function in sub-confluent cells (figure S1) and in cells exposed to 2% serum (figure S2). However, variant glucose exposure did not alter functional parameters under these conditions. Interestingly FCCP, at the concentration optimized for use in confluent cells, had much less effect in sub-confluent cells. Thus respiration was not maximized and we do not show data for those assessments. However, glucose concentration had no effect on OCR in sub-confluent cells after FCCP addition. Glucose also had no effect on OCR in confluent cells after FCCP administration when FCCP was injected with no prior oligomycin injection (figure S3).

Pyruvate, which is not generally included in Seahorse incubation medium, could impact the effects of variant glucose exposure. However, when supplemental pyruvate was included, we again saw no differences in the effects of added glucose on OCR; although pyruvate *per se* did increase ECAR (figure S4).

### Power to Detect Negative Effects on Glucose on OCR

**Figure 2:**
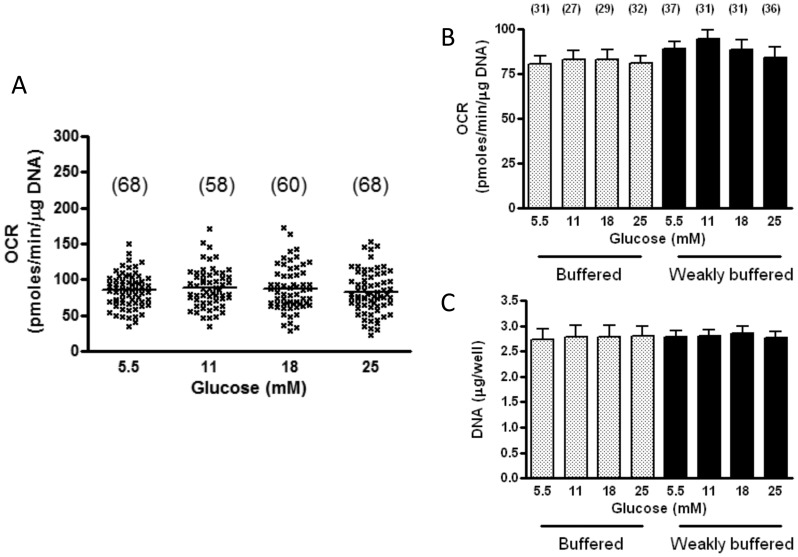
Effect of glucose on oxygen consumption rates (OCR) in BAE cells during basal incubation. Cells were grown to confluency and exposed overnight (18 h) to the glucose concentrations indicated. Respirometer experiments were carried out in weakly buffered (phosphate) medium as recommended by the manufacturer (Seahorse, Inc) for simultaneous detection of OCR and ECAR or in the same medium buffered with 25 mM HEPES. A) Basal OCR, all data (scatterplot with median value); B) Basal OCR (mean ± SE) in cells incubated in buffered or weakly buffered medium; C) DNA content per well (mean ± SE) in cells depicted in panel B. Glucose had no significant effects on OCR or DNA whether analyzed by ANOVA or multiple t-tests. Incubation in buffered compared to weakly buffered medium had no significant effects at any glucose concentration. Numbers in parentheses indicate number of repetitions.

Figure 2A provides a visual representation of power to detect a difference in our major outcome variable, the effect of overnight glucose exposure on basal OCR. This measure was repeated in 68 individual wells for glucose at 5.5, in 68 wells for glucose at 25 mM, and with only slightly less repetitions for glucose at 11 and 18 mM. These numbers include measurements made in both buffered (HEPES and phosphate) and weakly buffered (buffered with phosphate alone - as used to measure simultaneous ECAR and OCR) medium, since there was no difference according to buffering capacity (figure 2B). Differences between glucose at 5.5 mM in figure 2A were not significant in comparison to any of the elevated glucose groups whether analyzed by unpaired t-tests or ANOVA with multiple comparisons to control. These numbers provide a power of 95% to detect a 20% difference in means for 5.5 versus 25 mM by unpaired t-test at the observed standard deviation (32% of the mean) and a 78% power to detect a 15% difference in means. Moreover, since we also compare 18 and 11 mM glucose to 5.5 mM our power can be viewed as considerably greater to at least detect an effect of some degree of glucose elevation. Finally, we point out that similar power consideration apply beyond basal OCR, since we carried out the same number of measurements for data obtained on sequential addition of oligomycin, FCCP, and antimycin plus rotenone.

### Elevated Medium Glucose does not Alter Cell DNA and Mitochondrial Protein Content

Figure 2C shows that 18 h exposure to differing glucose concentrations had no effect on DNA content per well used in the Seahorse assays. In smaller numbers of samples, protein content was likewise unaffected and DNA to protein ratios remained constant in spite of varying antecedent glucose (figure S5). As indices of mitochondrial numbers, we assessed the cellular content of mitochondrial specific proteins normalized to total cell DNA and protein. The content of porin and complex IV protein were unaffected by medium glucose (figure S5); this figure also demonstrating that the outer membrane protein, porin, is, in fact, specifically expressed by mitochondria in BAE cells.

### Effect of Acute Glucose Exposure on OCR and ECAR after Antecedent (Overnight) Glucose Deprivation

**Figure 3:**
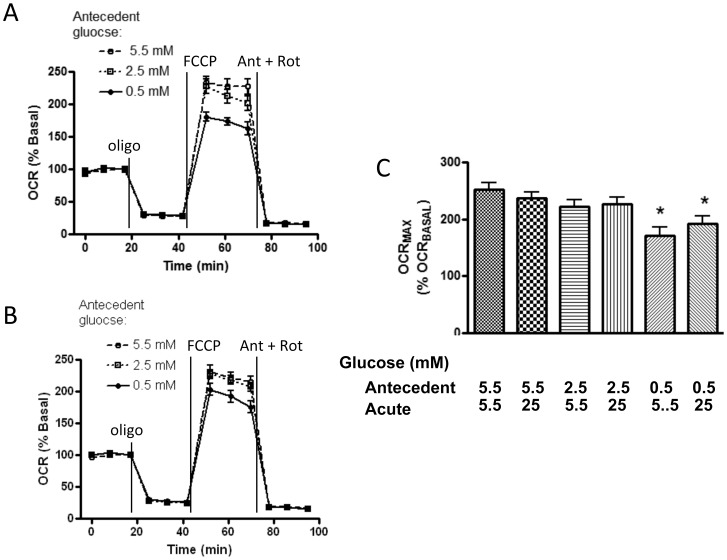
Effect of antecedent low glucose on mitochondrial bioenergetics in BAE cells. A) Mitochondrial function was assessed after 18 h exposure to 5.5, 2.5, or 0.5 mM glucose followed by acute exposure to 5.5 mM glucose. Data are expressed as percent basal (relative to the last basal data point just prior to addition of oligomycin) and represent mean ± SEM values determined in 12–16 wells. B) Mitochondrial function assessed after 18 h exposure to 5.5, 2.5, or 0.5 mM glucose followed by acute exposure to 25 mM glucose. Data points and injected compounds are as in panel A. C) Quantitative data comparing OCR on FCCP as a function of basal OCR in cells previously and then acutely exposed to the glucose concentrations indicated. Values represent mean ± SEM values over 9–12 wells, * p<0.05 ** p<0.01 compared to antecedent 5.5, acute 5.5.

Figure 3A shows that antecedent 18 h exposure to 0.5 mM glucose or to 2.5 mM glucose, compared to cells exposed to antecedent 5.5 mM glucose, did not alter basal OCR upon subsequent acute administration of 5.5 mM glucose. Likewise OCR during oligomycin and non-mitochondrial OCR were unaffected. However, maximal OCR (during FCCP) was reduced in cells exposed to antecedent low (0.5 mM) glucose. There was a trend towards the same effect of antecedent low glucose on maximal OCR when subsequent acute glucose exposure was set at 25 mM rather than 5.5 mM (figure 3B). Quantitative data are shown in figure 3C. ECAR was not significantly altered by these manipulations (data not shown).

### Effect of Fatty Acid Administration on OCR and ECAR

**Figure 4:**
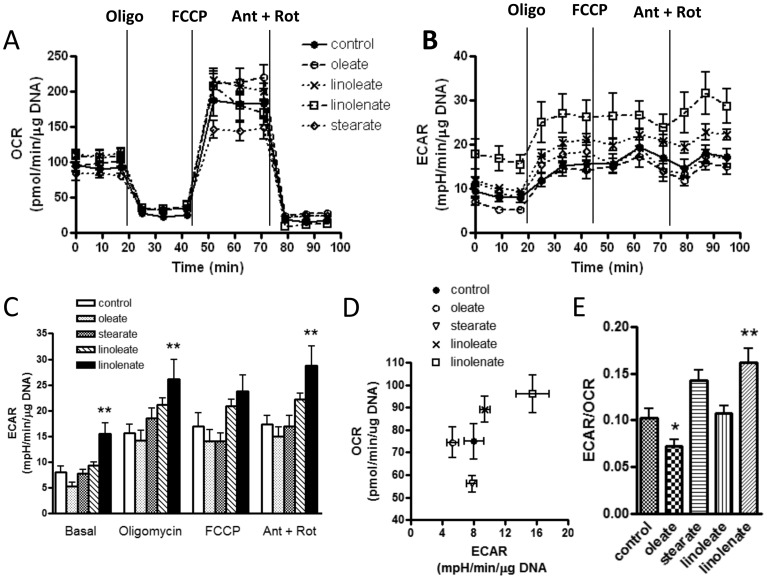
OCR and ECAR after overnight exposure to 18-carbon fatty acids of differing saturation. A) Oleate, stearate, linoleate, or linolenate did not alter OCR compared to control cells unexposed to fatty acid. Data represent mean ± SEM values at the time points indicated (n = 12–14 for each data point). B) ECAR determined simultaneously with OCR in panel A. Linolenate, the most highly unsaturated species, enhanced ECAR. C) Quantitative data (mean ± SEM ) comparing fatty acid effects on ECAR during the conditions indicated (Ant + Rot  =  antimycin A plus rotenone). D) OCR versus ECAR (mean ± SEM for both parameters) E) ECAR to OCR ratios according to fatty acid treatment (mean ± SEM). * p<0.05 or ** p<0.01 compared to control.

BAE cells were exposed to oleate at concentrations of 50 or 150 µM with 1.5% fatty acid-free BSA and 1 mM L-carnitine administered in both acute or overnight fashion (n = 12 to 14 wells per condition). These treatments had no significant effects on OCR or ECAR (data not shown). In additional experiments, cells were exposed for 18 h to 18-carbon fatty acids (150 µM) of differing saturation including oleate (18:1, n-9), stearate (18:0), linoleate, (18:2, n-6), or α-linolenate (18:3, n-3). These treatments did not alter OCR (figure 4A). However, ECAR was enhanced depending on the extent of unsaturation (figures 4B and 4C). We did note that stearate appeared partially toxic to cells which appeared partly fragmented. However, no such alterations were present after exposure to the other fatty acids. Linolenate enhanced both ECAR and OCR (figure 4D) and significantly increased the ratio of ECAR to OCR (figure 4E) while oleate decreased the ECAR to OCR ratio (figure 4E).

### Nutrient Oxidation to CO2 in BAE Cells Exposed to Elevated Medium Glucose

As an additional measure of nutrient oxidation in BAE cells, we measured CO_2_ production from labeled glucose, glutamine, and oleate in BAE cells exposed overnight to 5.5 or 25 mM glucose. In the presence of 20 µM oleate, treatment with 25 mM glucose compared to 5.5 mM glucose mildly, but significantly, increased glucose oxidation to CO_2_ by 7.4% (p<0.05, n = 12) and reduced oleate oxidation to CO_2_ by 10.3% (p<0.05, n = 12). In the presence of 2.7 mM glutamine (no added oleate), treatment with 25 mM glucose compared to 5.5 mM glucose did not alter glucose or glutamine oxidation to CO_2_ (n = 12_)_.

### ROS Production from BAE Cells Exposed to High Medium Nutrient Composition

**Figure 5:**
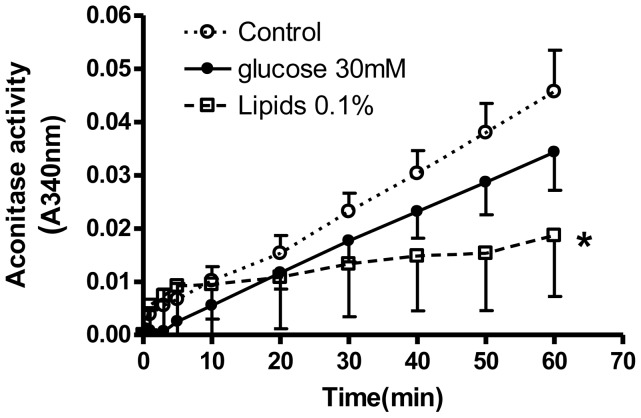
Aconitase activity in BAE cells exposed to antecedent glucose and lipid. Enzyme activity was determined in mitochondria isolated from BAE cells exposed to 5 mM glucose (control), 30 mM glucose, or 5 mM glucose plus 0.1% Intralipid (see text for composition) for 24 h. Activity was determined as NADPH production (A340nm) from cis-aconitate in the presence of exogenous isocitrate dehydrogenase monitored over 60 min. * p<0.05 compared to control by 2-way ANOVA, n = 6 per group.

We were unable to detect an increase in ROS production as oxidation of mitochondrial-targeted hydroethidine in cells exposed to 5.5 versus 25 mM glucose (figure S6). Addition of antimycin A as a positive control, well known to increase mitochondrial superoxide production [22], did increase HPLC peak intensity. We were also unable to detect an increase in H_2_O_2_ production from BAE cells exposed to high glucose (15, 20, or 25 mM) compared to 5.5 mM glucose for 24, 48, or 72. This was the case whether we used Amplex red or H2DCFDA as fluorescent probes (data not shown). We did observe a significant decrease in aconitase activity in cells exposed to high fat in the form of Intralipid for 24 h, but only a non-significant decrease in cells exposed to high glucose (figure 5). Intralipid is a fat emulsion designed for intravenous human administration as a nutrient supplement. It is largely made from soy bean oil and consists of linoleic (44–62%), oleic (19–30%), palmitic (7–14%), linolenic (4–11%) and stearic (1.4–5.5%) acids.

### Effect of Acute and Chronic Glycemia on Human Platelet Bioenergetics

**Figure 6:**
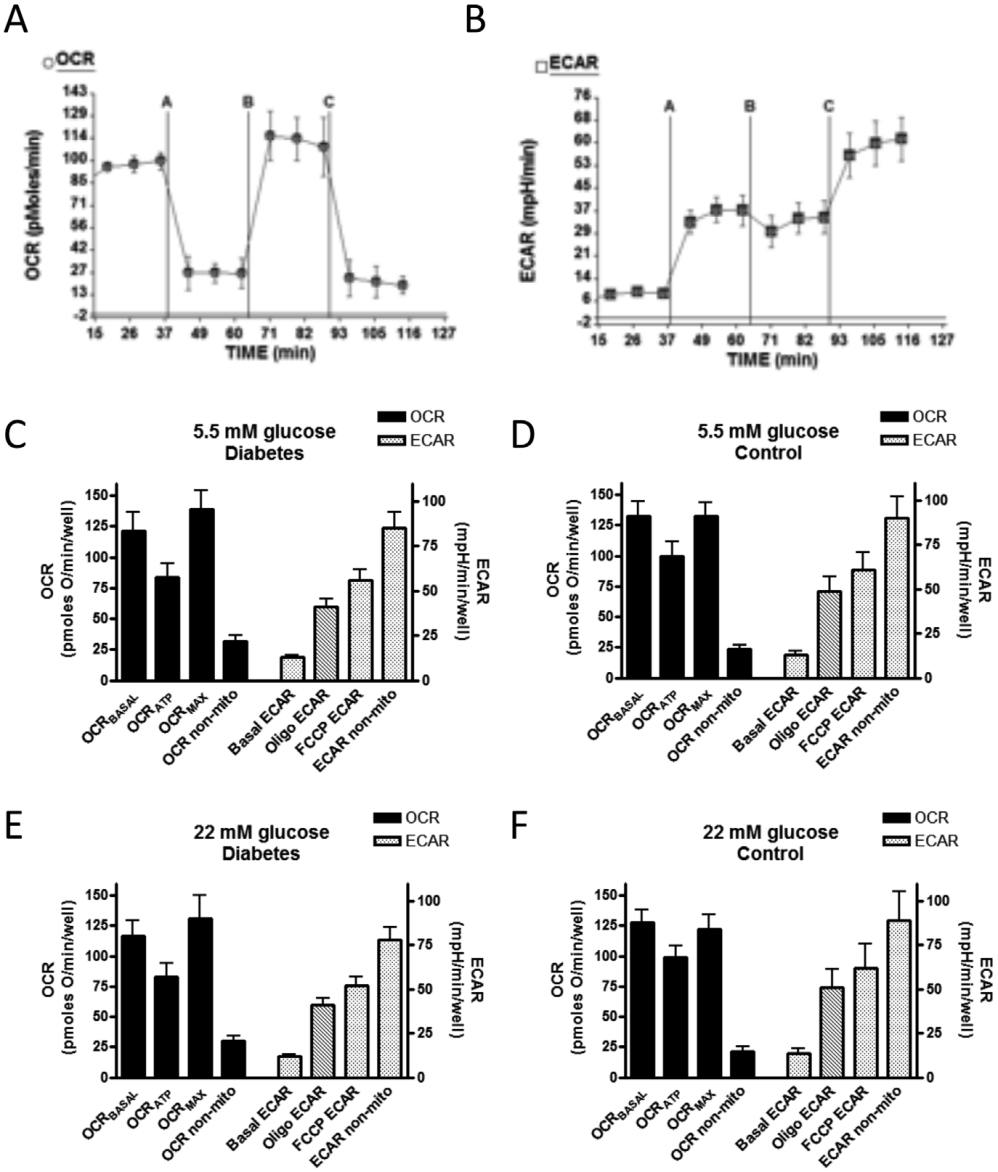
Effect of acute and antecedent (*in vivo*, before isolation) glucose exposure on human platelet mitochondrial function. Panels A and B) Representative tracings depicting OCR and ECAR by platelets (20×10^6^ per well) isolated from a healthy (non-diabetic) subject as affected by sequential additions of oligomycin (2 µM, injection point A), FCCP (2 µM, point B), and antimycin A (0.5 µM) plus rotenone (2 µM, point C). Each data point represents the mean ± SEM of 5 repetitions. Panels C-F) Calculated (see text) values for OCR_BASAL_, OCR_ATP_, OCR_FCCP_, and non-mitochondrial OCR and ECAR in diabetic and control subjects at 5.5 and 22 mM glucose (as specified in panel titles). Bars represent the mean ± SEM, n = 10 for diabetic subjects, n = 5 for control subjects. Each data point for each subject represents a mean of 4–5 repetitions.

Platelets were observed to adhere to the Seahorse well surface and remain in place throughout the assay procedure (figure S7). Incubating human platelets *in vitro* at 22 mM glucose, compared to 5.5 mM glucose, did not significantly alter OCR_BASAL_, OCR_ATP_, OCR_MAX_, or non-mitochondrial OCR whether the platelets derived from diabetic or control subjects (figure 6). Also, incubating platelets with 5.5 mM D-glucose plus 16.5 mM L-glucose (osmotic control) did not alter the above parameters in comparison to 5.5 or 22 mM D-glucose (data not shown). Moreover, these parameters did not differ between platelets obtained from subjects with type 1 diabetes and control subjects (figure 6). Likewise ECAR was not affected by these conditions (figure 6). Among the diabetic subjects, the OCR and ECAR parameters did not differ by gender (data not shown).

### Platelet Bioenergetics Compared to BAE Cells

**Figure 7:**
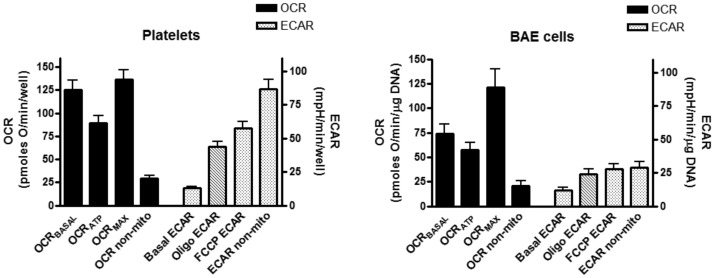
Comparison of mitochondrial bioenergetics between intact freshly isolated human platelets and BAE cells grown at 5.5 mM glucose. Glucose concentration was maintained at 5.5 mM during extracellular flux experiments. Platelet data represent mean ± SEM values for all 15 subjects that comprised the data in figure 6. BAE cell data represents 14 repetitions (mean ± SEM) for each data point.

Platelet mitochondria demonstrated robust mitochondrial oxidative metabolism in the basal state which was 4–5 fold greater than non-mitochondrial oxygen consumption (figure 7). Figure 7 contrasts OCR and ECAR between platelets and BAE cells. Based on the data in figure 7 (combining the entire group of diabetic and non-diabetic subjects), we calculated that OCR_BASAL_ was 93±5% of OCR_MAX_ in platelets, whereas OCR_BASAL_ in BAE cells was 65±4% of OCR_MAX_ (p<0.001 by unpaired t-test, n = 15 platelet and 14 BAE preparations). OCR_ATP_ accounted for 66±4% of maximal mitochondrial respiration in platelets compared to 51±4% in BAE cells (p<0.05). Calculated state apparent was 3.38±0.09 for platelet mitochondria compared to 3.69±0.06 for BAE cells (p<0.01). Absolute numbers for extent of oxygen consumption or acidification rate cannot be directly compared between endothelial cells and platelets since normalization was carried out differently (BAE cells to DNA content, platelet rates to number of platelets). However, extracellular acidification rates relative to OCR were clearly greater in platelets compared to BAE cells (figure 7).

## Discussion

Hyperglycemia is critical to the development of the microvascular complications of type 1 and type 2 diabetes and, at least in subgroups, to macrovascular complications as well [23], [24]. However, the mechanisms responsible are still debated. A decade ago it was posited [3], [25] that non-insulin-sensitive cells exposed to high circulating glucose are driven toward increased mitochondrial substrate oxidation and increased potential. This would, consequently, lead to greater mitochondrial ROS production and, ultimately, the complication of diabetes. Although those studies contributed substantially to generating interest in the role of ROS in diabetic complications, controversy is evident. The authors reported increased H_2_O_2_ production in bovine and aortic endothelial (BAE) cells exposed to high glucose in the medium and asserted that the increased ROS was due to glucose-driven increased mitochondrial substrate oxidation. That assertion was based on a citation of a study of insulinoma cells which generated over 2-fold more CO_2_ after high glucose exposure. BAE cells were not studied and we know of no other reports demonstrating high glucose driven increased mitochondrial substrate oxidation.

Here we used recently available methodology [10] to directly assess the effect of high nutrient exposure of intact BAE cells and platelets on mitochondrial oxygen consumption and extracellular acidification. We show that BAE cells as well as freshly isolated platelets remain robust in terms of maintaining constant mitochondrial oxidative metabolism in spite of widely variant glucose concentrations. This is true whether glucose exposure is acute or chronic (overnight) for the BAE cells and whether glucose exposure is acute or chronic (antecedent exposure to *in vivo* hyperglycemia) for the platelets. Moreover, this appears to hold for glucose added to confluent or sub-confluent cells, for cells exposed to antecedent low serum, and for cells studied in the presence of variant pyruvate in the medium.

Our data did reveal a slight increase in labeled glucose oxidation to CO_2_ by cells exposed to high glucose. However, this increase was marginal at best and only evident in the presence of oleate. Moreover, any such small increase in glucose-induced oxidation to CO_2_ may be offset by decreased oxidation of fat or other nutrients, such that there would be no overall increase in mitochondrial OCR. So, our findings for labeled glucose oxidation are consistent with data for OCR by extracellular flux.

Our major purpose was to determine whether elevated extracellular glucose enhances mitochondrial oxidation; an underlying supposition as to the reason why glucose might increase mitochondrial ROS production. As above we observed no such effect. Subject to limitations of the probes we used, we were also unable to detect any increase in whole cell ROS production in BAE cells exposed to high glucose either with 2,7-dichlorodihydrofluorescein diacetate (DCF) or 10-acetyl-3,7-dihydroxyphenoxazine (Amplex Red), two commonly used probes for hydrogen peroxide release (data not shown), or by oxidation of mitochondrial-targeted hydroethidine (figure S6). Thus, we were unable to confirm past reports of high glucose induced ROS in insulin-insensitive cells. Of note, the detection specificity for glucose-induced elevated H_2_O_2_ production reported in some past studies [2], [3] has been questioned [26], since those reports claimed inhibition by superoxide dismutase (or a mimetic), an enzyme that should increase (not decrease) H_2_O_2_ production. Further, although high glucose-induced ROS in insulin insensitive cells [1], [2], [3] and platelets [4] has been described in multiple reports, the concept is not supported by all studies. For example cultured hepatocytes exposed to high glucose generate more glycogen rather than increase respiration or potential [27] and some studies of non-insulin sensitive cells do not support an effect of glucose to induce ROS at the cell level [5], [6]. Differences may be due to methodology; including specific cell type(s) examined, antecedent cell nutrition, and the particular means of detecting ROS. We did detect a decrease in aconitase activity in BAE cells exposed to high glucose or fatty acids (figure 5); the enzyme serving as a marker for oxidative damage and representing a mitochondrial protein particular sensitive to oxygen radical-induced dysfunction [28]. However, this was statistically significant only for cells exposed to high fat in the medium, not for high glucose.

We note that Inoguchi et. al. [29] used a specific electron paramagnetic resonance technique to document increased ROS production from cultured aortic endothelial cells and smooth muscle cells. However, that those investigators concluded that the ROS derived from NAD(P)H oxidase and, thus, not expected to depend on mitochondrial oxidation or superoxide generated by electron transport.

We also show that oxidative metabolism by human platelets is not altered by antecedent exposure to high circulating glucose (as manifest in subjects with type 1 diabetes) or by acute *in vitro* exposure to high glucose. We are aware of only one very recent report describing platelet bioenergetics using the (Seahorse) extracellular flux approach [30]. That study examined platelets from small numbers of subjects with type 2 diabetes compared to controls and reported reduced basal OCR, maximal OCR on dinitrophenol (DNP), and OCR directed at ATP synthesis. ECAR was not reported. Our data contrast in that we did not see reduced respiration in our diabetic samples. Our subjects had type 1, not type 2, diabetes controlled on insulin and likely did not have the associated vascular risk often seen in type 2 diabetes. Our data confirm the feasibility of using the extracellular flux analyzer for platelet studies; an issue of some importance given that platelets represent an easily assessable source of human tissue for mitochondrial studies. In fact, other than biopsy material, circulating blood is the only available source. Moreover platelets, as opposed to neutrophils or lymphocytes, are far easier to prepare as fresh preparations in sufficient amounts for study. We also observed that platelets adhere strongly to wells within the extracellular flux analyzer throughout the assay procedure.

Oxidative metabolism by platelets has been subject to some debate since some reports claim that glucose use is primarily through non-oxidative glycolysis [31], [32], [33]. But, although devoid of nuclei, platelets do have mitochondria, so our data demonstrating mitochondrial oxidation does not seem surprising. It is also of note that platelets respired closer to maximal capacity than BAE cells, wherein maximal respiratory capacity far exceeded basal. In addition it is of interest that, relative to OCR, platelets manifest higher ECAR values compared to BAE cells (figure 7), consistent with a greater extent of non-oxidative glycolytic metabolism.

A prior report (which did not assess glucose or fatty acid effects) examined the bioenergetics of BAE cells using the extracellular flux approach [16]. Interestingly our data for state apparent of the BAE cells, 3.69 (see text under “results”), agrees closely with the value of 3.67 reported in the above citation.

Our studies revealed some additional findings of interest. Upon acute glucose administration after overnight low glucose, we found that maximal mitochondrial respiration was reduced in BAE cells (figure 3). This may have occurred due to overall antecedent cellular depletion of ATP. Respiration measured in the presence of FCCP is intended to assess “maximal respiration” in fully uncoupled mitochondria. However, respiration during FCCP in intact cells can reflect cytoplasmic factors such as ionophoric effects on endosomes and alterations in cytoplasmic calcium, thereby affecting substrate supply [9]. So, we can at least speculate that this effect, along with antecedent low glucose, limited substrate supply so that FCCP may not have had a true “maximal” action. In the regard, the reduction in “maximal” OCR after antecedent low glucose appeared less after acute 25 mM glucose than after 5.5 mM glucose (figure 3), suggesting that the higher acute glucose may have partially offset the depletion in energy stores.

We also noted that linolenic acid, the most unsaturated of the agents tested, enhanced ECAR and the ratio of ECAR to OCR in endothelial cells (figure 4), suggesting an increase in glycolytic metabolism to lactate. Plotting ECAR versus OCR revealed an upward and right shift for linolenic acid (figure 4D) suggesting a degree of uncoupling as well. In fact, there were trends in these directions for linoleic acid; the next most saturated fatty acid. Interestingly, oleate reduced the ratio of ECAR to OCR largely due to a reduction in ECAR possibly consistent with more relative oxidative metabolism but at a lower energetic state. It is possible to speculate that since linolenate is a relatively poor substrate for mitochondrial β-oxidation compared to more saturated fatty acids, the cells would rely more on glucose for ATP; thus, the higher ECAR. But if this were the case, we might expect ECAR in the presence of linolenate to become equivalent to that of the other fatty acids during mitochondrial inhibition (antimycin plus rotenone). Of course, there are other possible mechanisms including a myriad of effects that might result from alterations in gene expression.

There are limitations to our results. Although, our data show that excess glucose does not drive mitochondrial oxidation, we have only limited information as to the fate of excess glucose. In the BAE cells exposed to overnight high glucose, some portion is likely consumed through non-oxidative glycolysis generating lactate since ECAR was enhanced by glucose exposure. Platelets are known to store glycogen so glucose may have consumed in that process. We were unable to show an increase in ECAR in platelets exposed to high glucose suggesting that non-oxidative glycolysis was not enhanced. Of course, it is possible that the excess glucose provided to our cells or platelets simply was not transported across the plasma membrane and/or limited as to further metabolism after transport. The probes we used for detection ROS may not be specific for any one radical and we cannot be sure of the nature of the hydroethidine oxidation we observed without further assessment of the products eluted on HPLC. Moreover, competition by cellular superoxide dismutase and/or other antioxidant steps could mitigate the extent of probe detection. Finally, we did not measure platelet activation. Possibly, this differed between diabetic and non-diabetic subjects or differed as a result of glucose exposure *in vitro* and could conceivably alter oxygen use. But, this does not change the fact that we observed no difference in mitochondrial OCR or ECAR.

In summary, our major results show that acute and chronic high glucose or fatty acid exposure does not alter mitochondrial oxygen consumption by cultured BAE cells. Moreover, acute and antecedent *in vivo* high glucose exposure does not alter mitochondrial oxygen consumption by freshly isolated human platelets. Hence, our data do not support the concept that increased glucose driven mitochondrial electron transport is responsible for enhanced ROS production, suggesting that the diabetic milieu affects redox status in other ways. We contrast the bioenergetics of platelets to endothelial cells and show that platelets respire at closer to maximal capacity and manifest greater extracellular acidification than BAE cells (consistent with greater glycolytic metabolism). Further, we provide data suggesting that the extent of fatty acid saturation may enhance glycolysis without affecting oxidative metabolism.

## Supporting Information

Figure S1 **Effect of glucose on oxygen consumption rates (OCR) and extracellular acidification rates (ECAR) in sub-confluent BAE cells.** Cells were grown in the usual fashion (“methods”, main manuscript) for one day after seeding. Sub-confluent cells were then exposed to glucose concentrations ranging from 5.5 to 25 mM or to 5.5 mM D-glucose +19.5 mM L-glucose (5.5+L) for 18 h (overnight) prior to the respirometer studies. Glucose concentrations were maintained during incubation in the extracellular flux analyzer. Glucose exposure had no significant effects on OCR or ECAR under these conditions. n = 7–8 determinations at each glucose concentration. OCR and ECAR were determined before and after sequential injections of oligomycin (2 µM), FCCP (2 µM), or antimycin A (0.5 µM) plus rotenone (2 µM) as described under “methods”, main manuscript. Data for OCR in the presence of FCCP is not included. This is because FCCP actually reduced or did not change OCR relative to basal conditions, indicating the lack of optimization of the FCCP concentration for the sub-confluent condition. However, glucose had no effect to alter OCR after FCCP.
(TIF)

Figure S2 **Effect of glucose on oxygen consumption rates (OCR) and extracellular acidification rates (ECAR) in BAE cells exposed to low serum concentration.** Cells grown in the usual fashion (“methods”, main manuscript) except that three days after seeding, the medium was changed from 17% serum (used in other studied reported in this manuscript) to 2%. Cells were studied in the respirometer 24 h after the reduction in serum. Cells were exposed to glucose concentrations ranging from 5.5 to 25 mM or to 5.5 mM D-glucose +19.5 mM L-glucose (5.5+L) for 18 h prior to the respirometer studies with these concentrations maintained during incubation in the extracellular flux analyzer. n = 4 determinations at each glucose concentration. OCR and ECAR were determined before and after sequential injections of oligomycin (2 µM), FCCP (2 µM), and antimycin A (0.5 µM) plus rotenone (2 µM) as described under “methods”, main manuscript. Glucose exposure had no significant effects on OCR or ECAR under these conditions.
(TIF)

Figure S3 **Effect of glucose on oxygen consumption rates (OCR) in BAE cells during basal incubation and in the presence of FCCP added either before or after oligomycin.** Confluent cells were exposed to glucose concentrations (mM) as shown on the x-axes ranging from 5.5 to 25 mM or to 5.5 mM D-glucose plus 19.5 mM L-glucose (5.5+L) for 18 h prior to the respirometer studies. Basal OCR (panel A) is depicted in cells subsequently treated with oligomycin (dotted bars) or FCCP (filled bars) (panel B). Respirometer incubations were carried out under basal conditions for 21 min followed by oligomycin (2 µM) or FCCP (2 µM) for 21 min. Cells treated with oligomycin were then exposed to FCCP (2 µM) for an additional 21 min. OCR measurements were taken at the end of each time period. Glucose did not significantly alter OCR under these conditions. n = 4–5 determinations at each glucose concentration.
(TIF)

Figure S4 **Effect of glucose and variant pyruvate concentration on oxygen consumption rates (OCR) and extracellular acidification rates (ECAR) in confluent BAE cells.** Cells were grown in the usual fashion (“methods”, main manuscript) and studied under basal conditions (panels A and B), in the presence of oligomycin (panels C and D), and in the presence of FCCP (panels E and F). Pyruvate was added acutely during the respirometer runs at the concentrations shown. Cells were exposed for 18 h to the glucose concentrations indicated with these glucose concentrations maintained during incubation in the extracellular flux analyzer. n = 4–6 determinations for each condition. Pyruvate had no significant effects on OCR but had a significant overall effect on ECAR (p<0.01) by two-way ANOVA (glucose x pyruvate x interaction) for each condition (basal, oligomycin, and FCCP). Glucose and interaction were not significant. * p<0.05 versus 0.1 mM pyruvate by Bonferroni posttests.
(TIF)

Figure S5 **Total and specific mitochondrial protein content per unit DNA is not altered by overnight exposure to high glucose concentration.** Cells were grown to confluency as described under “methods”, main manuscript. Panel A) Porin content in mitochondrial (M), whole cell (W), and supernatant (cytoplasmic) (S) BAE cell fractions. Data is representative of 4 repetitions of this blot demonstrating that BAE cell porin is localized to mitochondria. Panels B and C) Representative blots depicting porin and mitochondrial complex 4 protein (OxPhos) at expected kD (arrows) in whole BAE cells grown to confluency and exposed to 5.5 or 25 mM glucose for 18 h before preparation of cell extracts. Panels D to E) Quantification of porin and OxPhos as a function of whole cell protein. Panel F) DNA/protein ratios in the same cell extracts. Panels G and H) Quantification of porin and OxPhos protein as a function of whole cell DNA. Glucose exposure did not significantly alter these parameters. n = 8–10 repetitions for each group.
(TIF)

Figure S6 **Glucose does not alter oxidation of MitoSOX in BAE cells.** Cells were grown to confluency, then exposed overnight (18 h) to 5.5 or 25 mM glucose. Some cells exposed to 5.5 mM glucose were then treated with Antimycin A (10 µM) for 60 min. HPLC was carried out as described in “methods”, main manuscript. Upper tracings in each panel depict detection by UV. Lower tracings depict fluorescence. The major peak, eluting at 15.6 minutes, was enhanced by Antimycin A, a positive control, known for its strong induction of mitochondrial superoxide through action on Coenzyme Q redox cycling in Complex III. A) Cells exposed to 5.5 mM glucose; B) 25 mM glucose or; C) 5.5 mM glucose then Antimycin A. D) Quantitative data. *p<0.01 compared to 5.5 mM glucose by one-way ANOVA and Dunnett’s posttest; n = 8 for each glucose concentration, n = 3 for antimycin A.
(TIF)

Figure S7 **Platelets adhere to respirometer plates.** Platelets were imaged by light microscopy after Seahorse runs (as performed in figure 6, main manuscript). A) Platelets exposed to 5.5 mM glucose B) Platelets exposed to 25 mM glucose. Magnification x 40. Each panel is representative of 3 repetitions.
(TIF)
