# Supplementary figures and images for: Endothelial Cell and Platelet Bioenergetics: Effect of Glucose and Nutrient Composition
Source: PLoS One. 2012 Jun 22;7(6):e39430. doi: 10.1371/journal.pone.0039430 (PMC3382132; doi:10.1371/journal.pone.0039430)

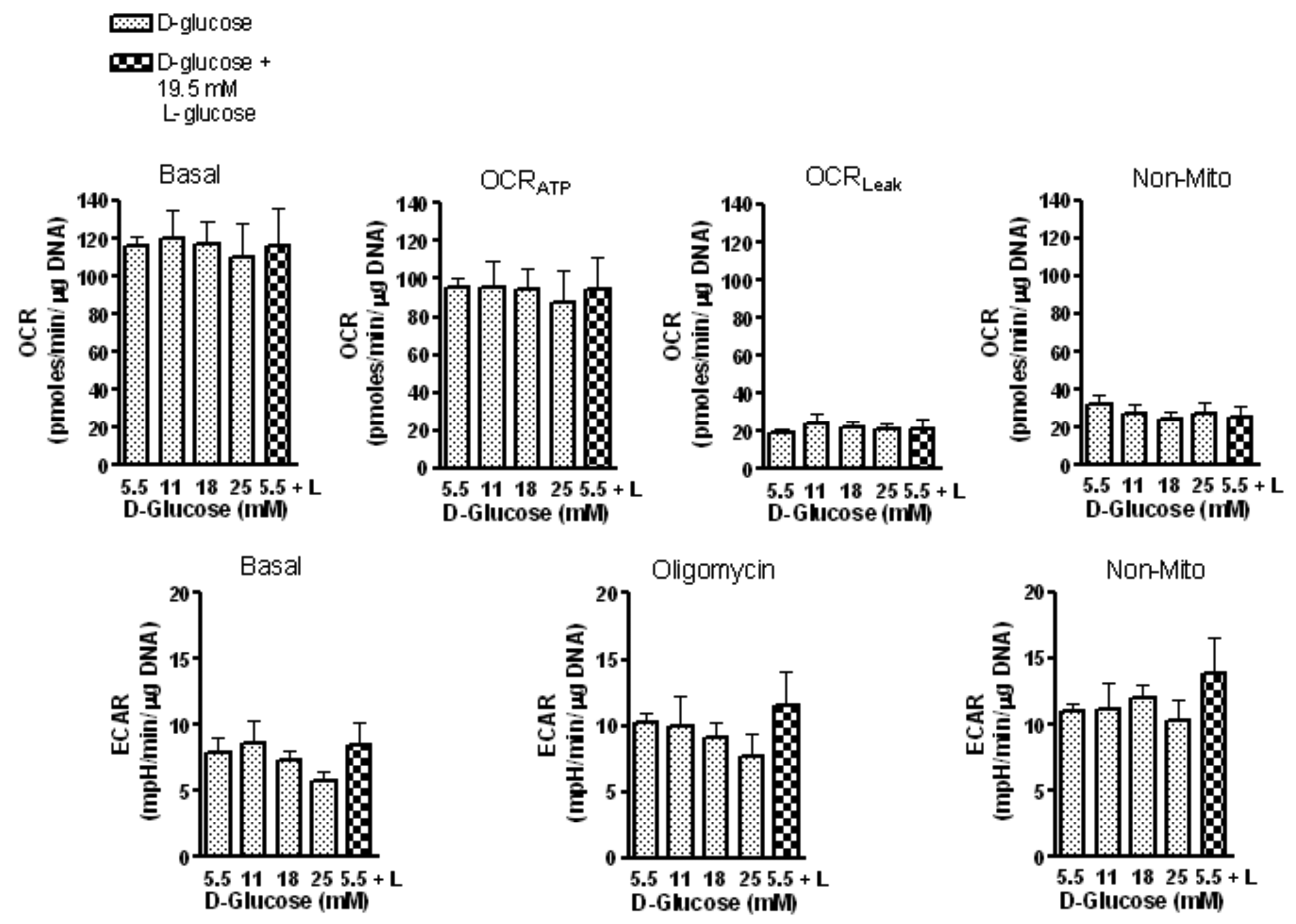

Supplement: Figure S1 — Effect of glucose on oxygen consumption rates (OCR) and extracellular acidification rates (ECAR) in sub-confluent BAE cells. Cells were grown in the usual fashion (“methods”, main manuscript) for one day after seeding. Sub-confluent cells were then exposed to glucose concentrations ranging from 5.5 to 25 mM or to 5.5 mM D-glucose +19.5 mM L-glucose (5.5+L) for 18 h (overnight) prior to the respirometer studies. Glucose concentrations were maintained during incubation in the extracellular flux analyzer. Glucose exposure had no significant effects on OCR or ECAR under these conditions. n = 7–8 determinations at each glucose concentration. OCR and ECAR were determined before and after sequential injections of oligomycin (2 µM), FCCP (2 µM), or antimycin A (0.5 µM) plus rotenone (2 µM) as described under “methods”, main manuscript. Data for OCR in the presence of FCCP is not included. This is because FCCP actually reduced or did not change OCR relative to basal conditions, indicating the lack of optimization of the FCCP concentration for the sub-confluent condition. However, glucose had no effect to alter OCR after FCCP. (TIF) [file pone.0039430.s001.tif]

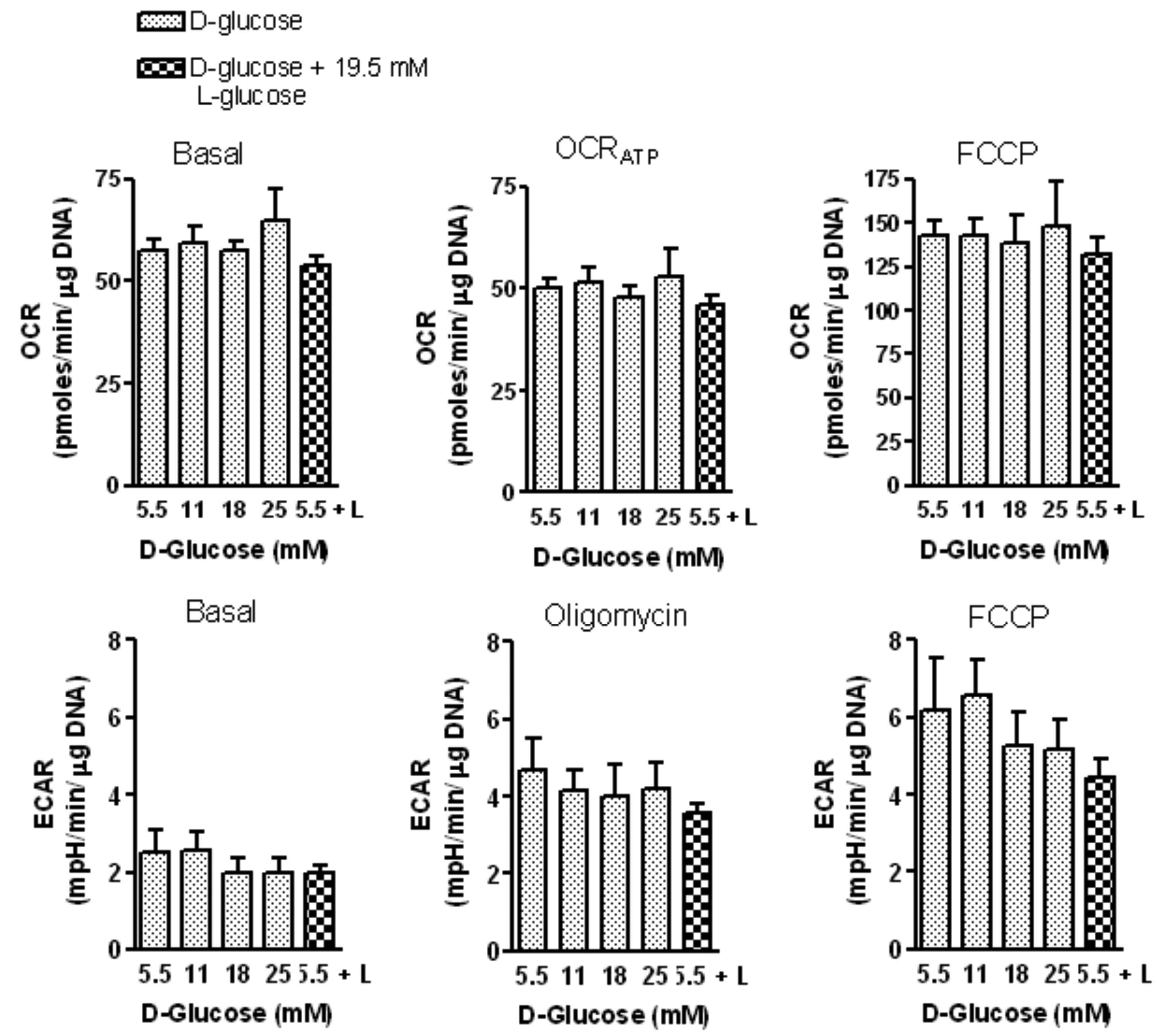

Supplement: Figure S2 — Effect of glucose on oxygen consumption rates (OCR) and extracellular acidification rates (ECAR) in BAE cells exposed to low serum concentration. Cells grown in the usual fashion (“methods”, main manuscript) except that three days after seeding, the medium was changed from 17% serum (used in other studied reported in this manuscript) to 2%. Cells were studied in the respirometer 24 h after the reduction in serum. Cells were exposed to glucose concentrations ranging from 5.5 to 25 mM or to 5.5 mM D-glucose +19.5 mM L-glucose (5.5+L) for 18 h prior to the respirometer studies with these concentrations maintained during incubation in the extracellular flux analyzer. n = 4 determinations at each glucose concentration. OCR and ECAR were determined before and after sequential injections of oligomycin (2 µM), FCCP (2 µM), and antimycin A (0.5 µM) plus rotenone (2 µM) as described under “methods”, main manuscript. Glucose exposure had no significant effects on OCR or ECAR under these conditions. (TIF) [file pone.0039430.s002.tif]

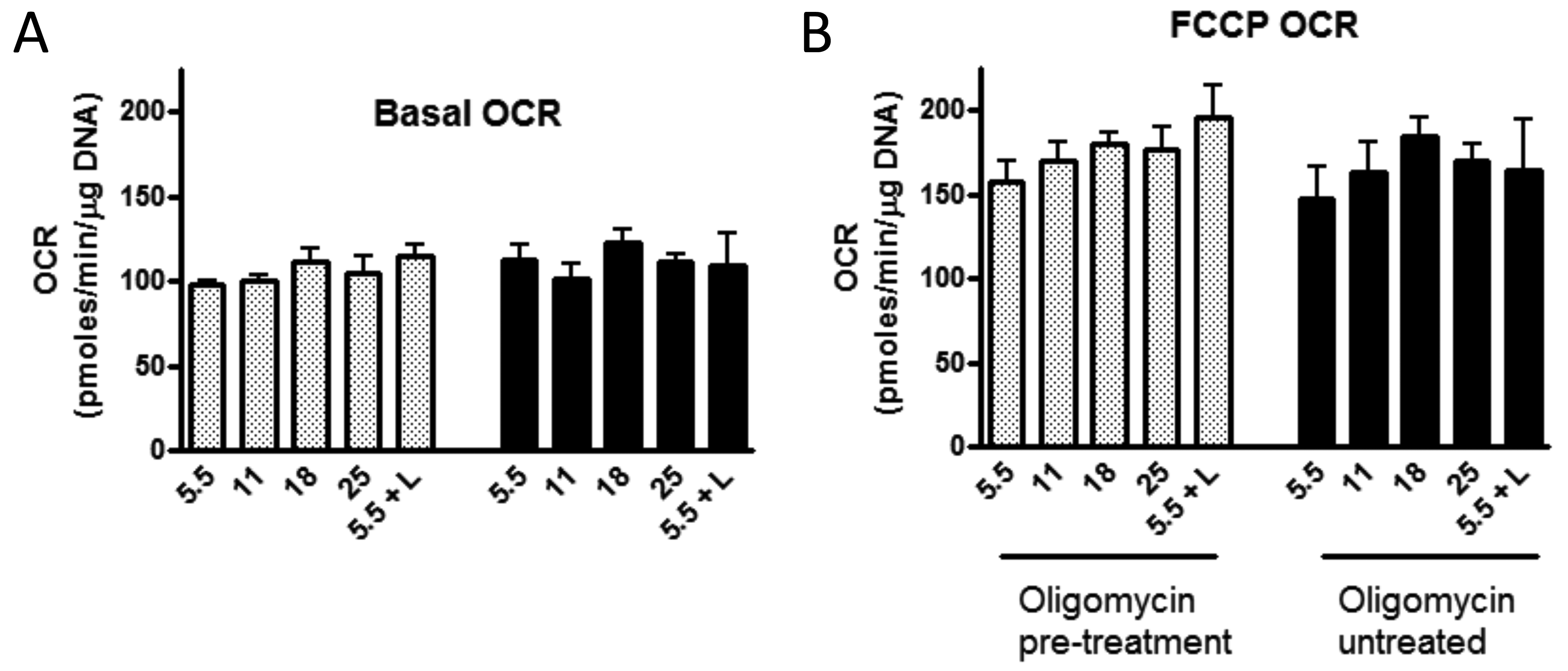

Supplement: Figure S3 — Effect of glucose on oxygen consumption rates (OCR) in BAE cells during basal incubation and in the presence of FCCP added either before or after oligomycin. Confluent cells were exposed to glucose concentrations (mM) as shown on the x-axes ranging from 5.5 to 25 mM or to 5.5 mM D-glucose plus 19.5 mM L-glucose (5.5+L) for 18 h prior to the respirometer studies. Basal OCR (panel A) is depicted in cells subsequently treated with oligomycin (dotted bars) or FCCP (filled bars) (panel B). Respirometer incubations were carried out under basal conditions for 21 min followed by oligomycin (2 µM) or FCCP (2 µM) for 21 min. Cells treated with oligomycin were then exposed to FCCP (2 µM) for an additional 21 min. OCR measurements were taken at the end of each time period. Glucose did not significantly alter OCR under these conditions. n = 4–5 determinations at each glucose concentration. (TIF) [file pone.0039430.s003.tif]

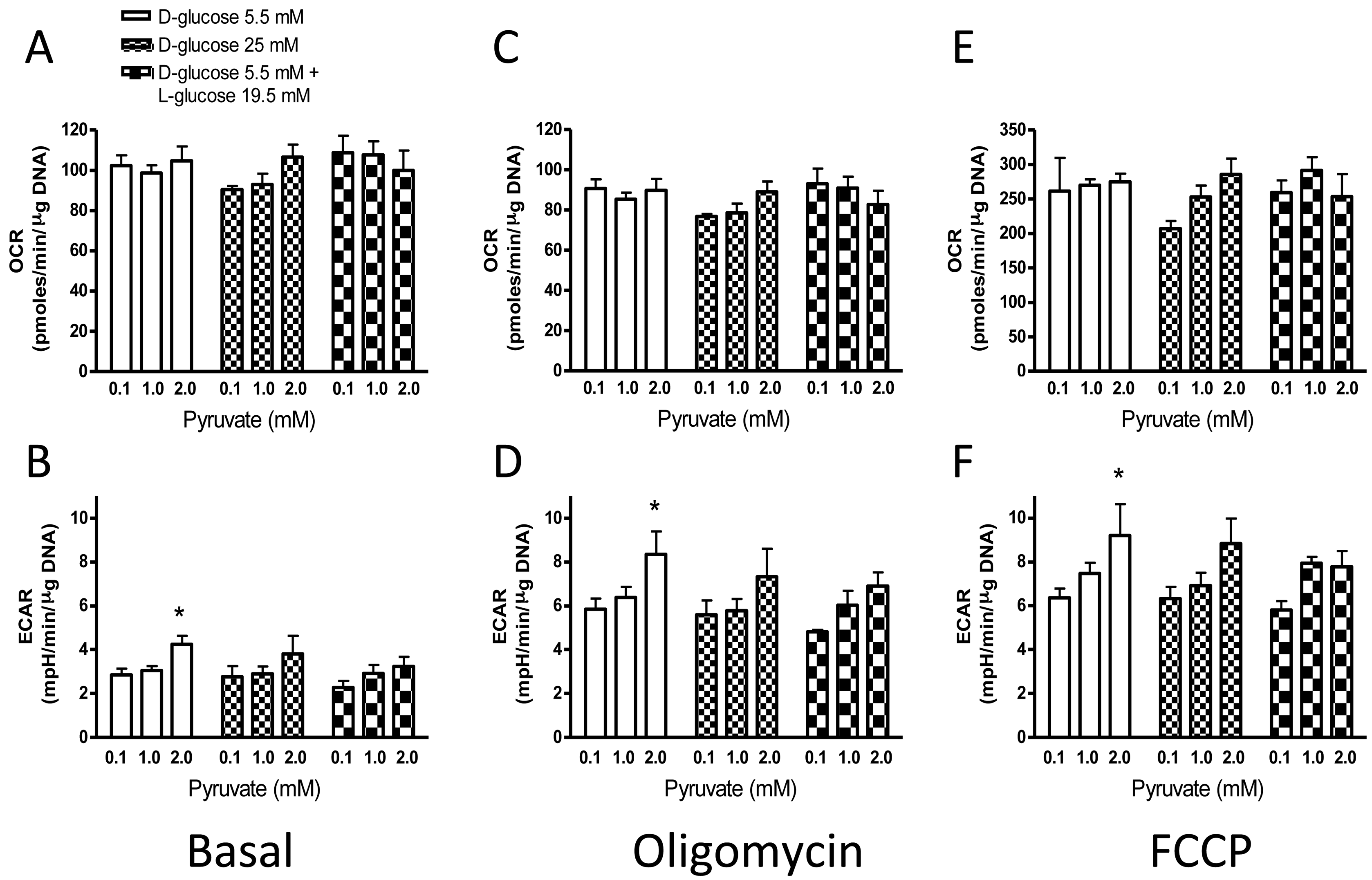

Supplement: Figure S4 — Effect of glucose and variant pyruvate concentration on oxygen consumption rates (OCR) and extracellular acidification rates (ECAR) in confluent BAE cells. Cells were grown in the usual fashion (“methods”, main manuscript) and studied under basal conditions (panels A and B), in the presence of oligomycin (panels C and D), and in the presence of FCCP (panels E and F). Pyruvate was added acutely during the respirometer runs at the concentrations shown. Cells were exposed for 18 h to the glucose concentrations indicated with these glucose concentrations maintained during incubation in the extracellular flux analyzer. n = 4–6 determinations for each condition. Pyruvate had no significant effects on OCR but had a significant overall effect on ECAR (p<0.01) by two-way ANOVA (glucose x pyruvate x interaction) for each condition (basal, oligomycin, and FCCP). Glucose and interaction were not significant. * p<0.05 versus 0.1 mM pyruvate by Bonferroni posttests. (TIF) [file pone.0039430.s004.tif]

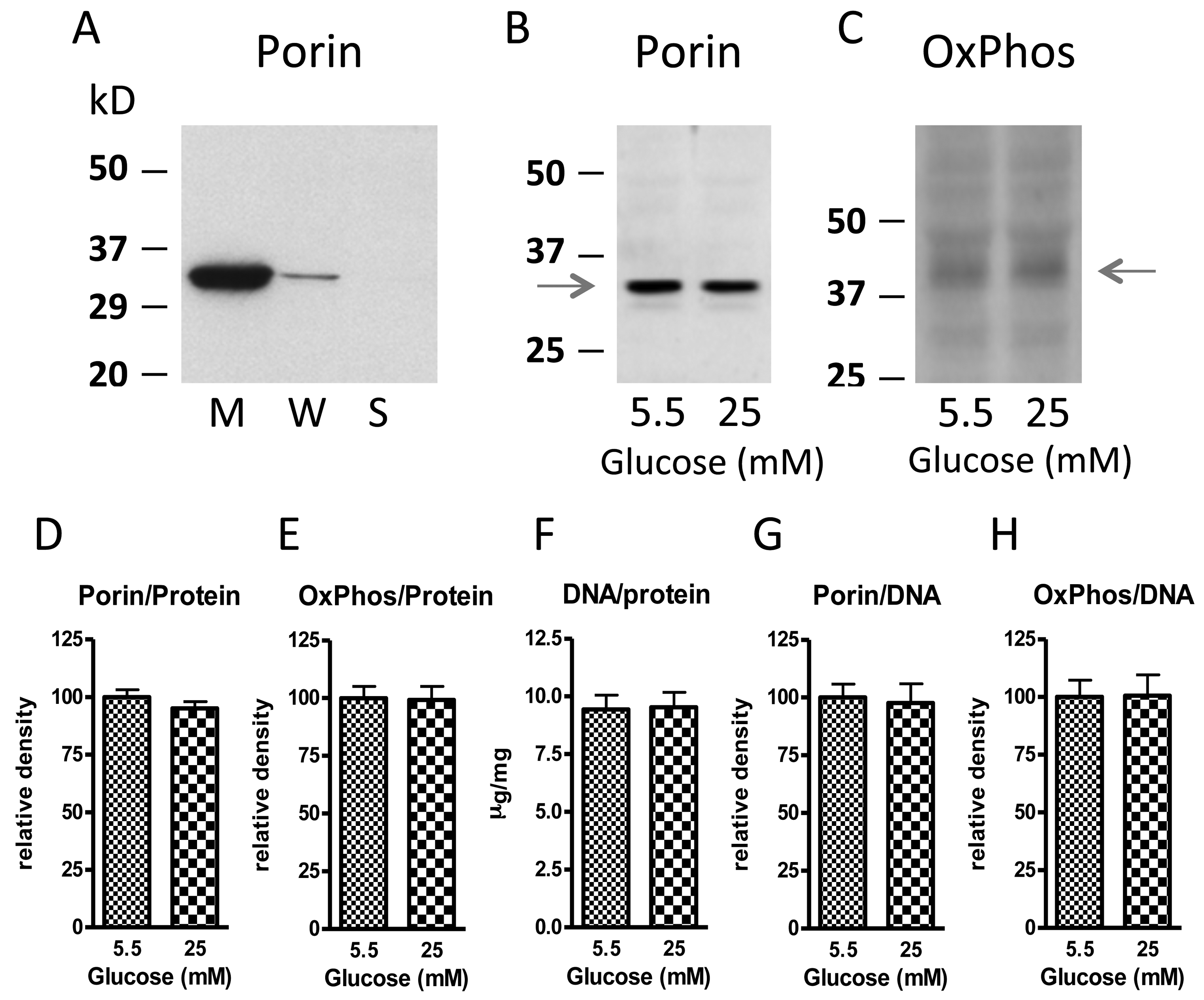

Supplement: Figure S5 — Total and specific mitochondrial protein content per unit DNA is not altered by overnight exposure to high glucose concentration. Cells were grown to confluency as described under “methods”, main manuscript. Panel A) Porin content in mitochondrial (M), whole cell (W), and supernatant (cytoplasmic) (S) BAE cell fractions. Data is representative of 4 repetitions of this blot demonstrating that BAE cell porin is localized to mitochondria. Panels B and C) Representative blots depicting porin and mitochondrial complex 4 protein (OxPhos) at expected kD (arrows) in whole BAE cells grown to confluency and exposed to 5.5 or 25 mM glucose for 18 h before preparation of cell extracts. Panels D to E) Quantification of porin and OxPhos as a function of whole cell protein. Panel F) DNA/protein ratios in the same cell extracts. Panels G and H) Quantification of porin and OxPhos protein as a function of whole cell DNA. Glucose exposure did not significantly alter these parameters. n = 8–10 repetitions for each group. (TIF) [file pone.0039430.s005.tif]

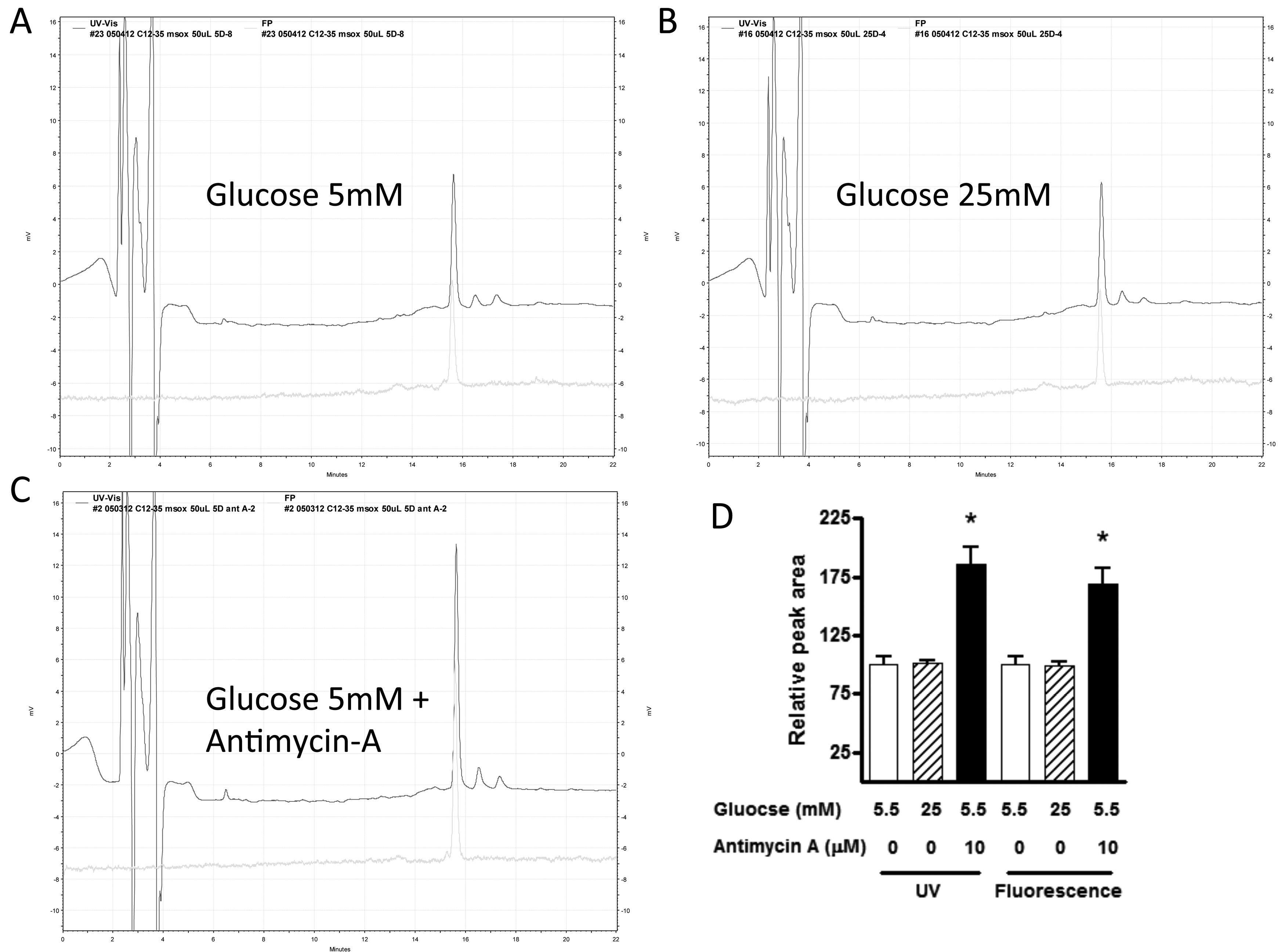

Supplement: Figure S6 — Glucose does not alter oxidation of MitoSOX in BAE cells. Cells were grown to confluency, then exposed overnight (18 h) to 5.5 or 25 mM glucose. Some cells exposed to 5.5 mM glucose were then treated with Antimycin A (10 µM) for 60 min. HPLC was carried out as described in “methods”, main manuscript. Upper tracings in each panel depict detection by UV. Lower tracings depict fluorescence. The major peak, eluting at 15.6 minutes, was enhanced by Antimycin A, a positive control, known for its strong induction of mitochondrial superoxide through action on Coenzyme Q redox cycling in Complex III. A) Cells exposed to 5.5 mM glucose; B) 25 mM glucose or; C) 5.5 mM glucose then Antimycin A. D) Quantitative data. *p<0.01 compared to 5.5 mM glucose by one-way ANOVA and Dunnett’s posttest; n = 8 for each glucose concentration, n = 3 for antimycin A. (TIF) [file pone.0039430.s006.tif]

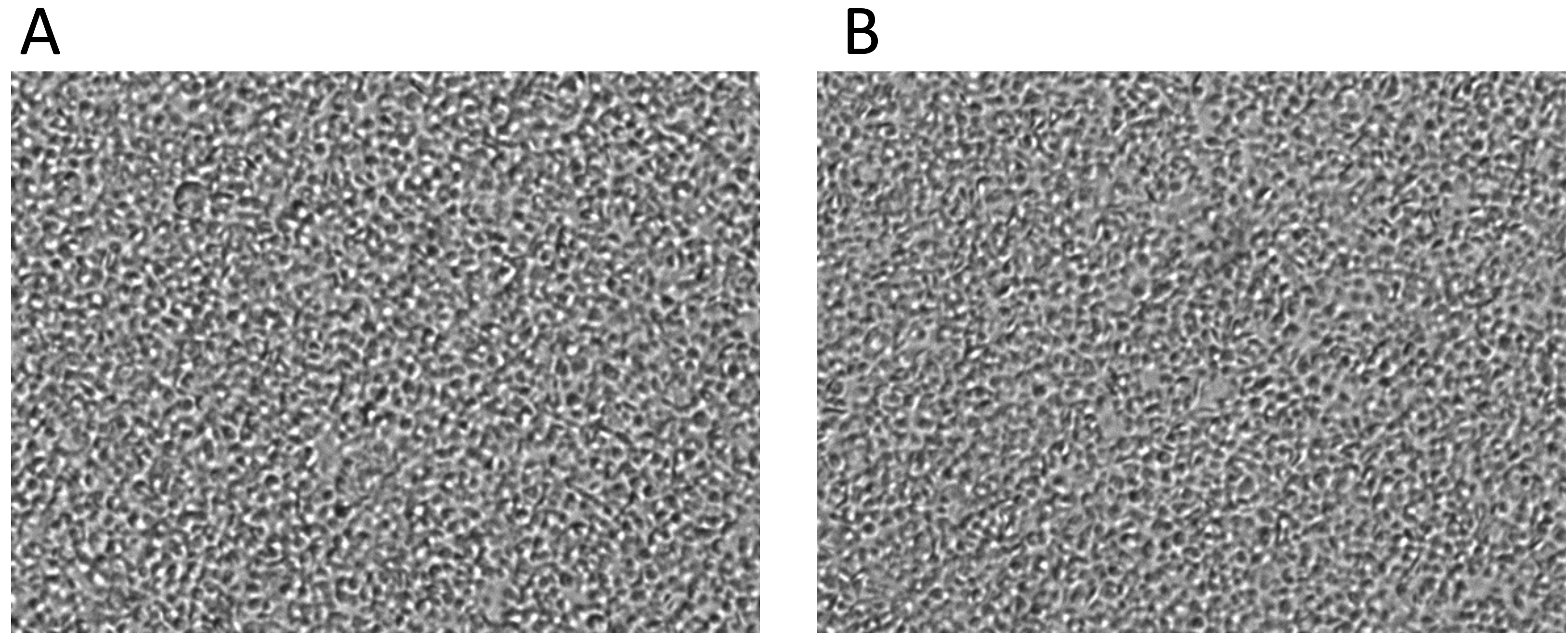

Supplement: Figure S7 — Platelets adhere to respirometer plates. Platelets were imaged by light microscopy after Seahorse runs (as performed in figure 6, main manuscript). A) Platelets exposed to 5.5 mM glucose B) Platelets exposed to 25 mM glucose. Magnification x 40. Each panel is representative of 3 repetitions. (TIF) [file pone.0039430.s007.tif]
